# Amending mine tailing cover with compost and biochar: effects on vegetation establishment and metal bioaccumulation in the Finnish subarctic

**DOI:** 10.1007/s11356-021-14865-8

**Published:** 2021-06-20

**Authors:** Marleena Hagner, Marja Uusitalo, Hanna Ruhanen, Juha Heiskanen, Rainer Peltola, Kari Tiilikkala, Juha Hyvönen, Pertti Sarala, Kari Mäkitalo

**Affiliations:** 1 Natural Resources Institute Finland (Luke), FI-31600, Jokioinen, Finland; 2 Ecosystems and Environment Research Programme, Faculty of Biological and Environmental Sciences, University of Helsinki, 15140 Lahti, Finland; 3 Natural Resources Institute Finland (Luke), FI-96200 Rovaniemi, Finland; 4 Natural Resources Institute Finland (Luke), FI-77600 Suonenjoki, Finland; 5 Present Address: KT-FinnoServ, FI-33180 Tampere, Finland; 6 Geological Survey of Finland (GTK), FI-96100 Rovaniemi, Finland; 7 Oulu Mining School (OMS), University of Oulu (Oulun yliopisto), FI-90014 Oulu, Finland

**Keywords:** Sewage sludge, Revegetation, Bioavailability, Tailing repositories, Carbon, Metal uptake

## Abstract

In the northern boreal zone, revegetation and landscaping of closed mine tailings are challenging due to the high concentrations of potentially toxic elements; the use of nutrient-poor, glacigenic cover material (till); cool temperatures; and short growing period. Recycled waste materials such as biochar (BC) and composted sewage sludge (CSS) have been suggested to improve soil forming process and revegetation success as well as decrease metal bioavailability in closed mine tailing areas. We conducted two field experiments in old iron mine tailings at Rautuvaara, northern Finland, where the native mine soil or transported cover till soil had not supported plant growth since the mining ended in 1989. The impacts of CSS and spruce (*Picea abies*)–derived BC application to till soil on the survival and growth of selected plant species (*Pinus sylvestris*, *Salix myrsinifolia*, and grass mixture containing *Festuca rubra*, *Lolium perenne*, and *Trifolium repens*) were investigated during two growing seasons. In addition, the potential of BC to reduce bioaccumulation of metals in plants was studied. We found that (1) organic amendment like CSS markedly enhanced the plant growth and is therefore needed for vegetation establishment in tailing sites that contained only transported till cover, and (2) BC application to till soil-CSS mixture further facilitated the success of grass mixtures resulting in 71–250% higher plant biomass. On the other hand, (3) no effects on *P. sylvestris* or *S. myrsinifolia* were recorded during the first growing seasons, and (4) accumulation of metals in cover plants was negligible and BC application to till further decreased the accumulation of Al, Cr, and Fe in the plant tissues.

**Graphical abstract:**

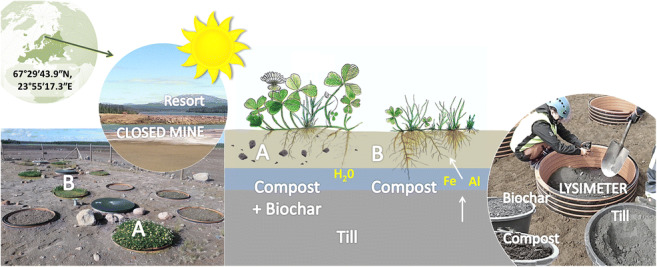

**Supplementary Information:**

The online version contains supplementary material available at 10.1007/s11356-021-14865-8.

## Introduction

In order to minimize the release of the harmful effluents from mine wastes into the immediate environment, closed mine tailings are usually covered with low permeability materials (Kauppila et al. 2013). According to international guidelines, mine sites must be landscaped not only after closing (i.e., rehabilitation) but also during production (EC 2012). A good tailing cover retains water, prevents erosion, increases evapotranspiration, and reduces oxygen flow to mine waste layers, reducing the oxidation and leaching of sulfide minerals into the environment (Lottermoser 2007). As vegetation enhances these processes, the rapid establishment of vegetation after covering is important. Vegetation also increases soil carbon content, which further favors natural vegetation succession.

The properties of tailings depend on the mineralogy and ore type of mine, and the enrichment process used to separate or leach out the metals from the ore as well as the volume of enrichment sand to be deposited on the site (Ndlovu et al. 2017). In this respect, the sulfide ore types are typically the worst due to their high acid production capacity and metal availability in the tailings, but also other types of ores can cause harm if the heavy metal contents are high (Ndlovu et al. 2017). Previously, the covering methodology consisted mainly of local till soil. Nowadays, the covering is planned for the local circumstances and depending on tailing properties and may consist for example of barrier layer (e.g., geomembrane), till soil, and vegetation layer (Heikkinen et al. 2008; Tuomela et al. 2021).

There are many challenges in revegetation of tailing repositories. Firstly, materials that are available for the cover material depend highly on a terrain and climate conditions where the mine sites are located. Due to long glacial history in the Northern Hemisphere, glacigenic sediments are the most dominant surface sediment types in all continents. For that reason, glacial till is still the most used cover material in old and present mine sites, for example in the Nordic countries. However, the till material usually does not favor vegetation success due to the generally low organic matter and nutrient contents and low pH (Krzaklewski and Pietrzykowski 2002; Tordoff et al. 2000). Plant establishment on the mine tailing repositories is impeded also by climatic factors including extreme temperatures, low precipitation, and heavy winds. Especially in the Arctic areas and boreal zone, cool temperatures and a short growing season restrict vegetation growth.

Secondly, the high concentrations of metals such as arsenic (As), cadmium (Cd), copper (Cu), lead (Pb), and zinc (Zn) in tailing repositories challenge the revegetation on mine tailings (Lottermoser 2007; Chileshe et al. 2020). The ability of various plants to take up, tolerate, and bioaccumulate different metals varies considerably (Filipović-Trajković et al. 2012; Reeves and Baker 2000). The reactions often depend on growth strategy of taxa, phase of growth, how much a heavy metal chemically resembles macronutrients, and availability of metals in soils (Grime 2001; Willey et al. 2005). The highest uptake of cesium (Cs), for example, is shown to be typical to stress-tolerant ruderal species that are first to colonize disturbed lands and exhibit poor discrimination between potassium (K) and Cs uptake (Grime 2001; Willey et al. 2005). By contrast, the lowest uptake is typical to species with a competitor strategy to maximize the capture of resources. Various grasses (e.g., Poaceae and *Festuca* spp.) belong to the latter group and are often used in landscaping of mine tailings due to their rapid growth and, consequently, ability to decrease erosion and retain water and nutrients (Koivuhuhta et al. 2019). In addition, pines have been suggested to be used due to their low bioaccumulation of metals (Jana et al. 2012). In contrast, the fast-growing woody species, e.g., *Salix* spp., are recommended for cleaning-up contaminated lands due to their high biomass-producing capacity (Masarovičová and Kráľová 2012).

Thirdly, transported covering materials have a marked effect on revegetation success in the tailing repositories. Recently, waste materials such as biochar (BC) and composted sewage sludge (CSS) have been suggested to improve landscaping, vegetation success, and carbon sequestration (Ali et al. 2017; Méndez et al. 2015) of highly degraded soils such as mine closures (Fellet et al. 2014; Peltz and Harley 2015; Penido et al. 2019). BC has been reported to enhance soil fertility by improving the physical, chemical, and biological properties of the cover material and retaining nutrients and water, thus providing valuable growing media for plants (Arif et al. 2017; Panwar et al. 2019). BC has also been proposed as a remediation strategy to increase soil pH and reduce the leaching of toxic elements (Beesley et al. 2011; Park et al. 2011; Penido et al. 2019). Several studies have demonstrated the effectiveness of BCs to decrease metal bioavailability. For example, Fellet et al. (2011) and Penido et al. (2019) reported decreased Cd, Pb, and Zn bioavailability in mine soils following the incorporation of an orchard waste– and eucalyptus (respectively)–derived BC. Also, hardwood-derived BC was reported to decrease concentrations of Cu in pore water and Cu and Pb concentrations in the shoots of ryegrass (*Lolium perenne*) grown in the mine soil in England (Karami et al. 2011).

Using BC with other additives in the repository cover layer might allow incorporation of a wider variety of plants in revegetation. For example, CSS contains abundant nutrients and organic matter. CSS is produced at over 10 million tonnes of dry solids per year in over 50,000 wastewater treatment plants within the European Union (Eurostat 2018). In the EU countries, 37% of the total production of sewage sludge is used in agriculture, the rest being used for other applications such as green building and landscaping (Olofsson et al. 2012). However, some sewage sludges can exceed the threshold values for metal concentrations (The Sewage Sludge Directive 86/278/EEC) and contain organic pollutants (Zennegg et al. 2013), unwanted pathogens (Bagge et al. 2005), and microplastics (Lusher et al. 2017), compromising their utilization.

There are only few reported field studies that have evaluated the vegetation success and metal bioaccumulation in plants in mine tailing repositories in the northern countries, where the short growing season and harsh winters restrict plant survival and growth. Even globally, most studies suggesting BC as an amendment for mine tailings have been performed under greenhouse or laboratory conditions (Ghosh and Maiti 2020). Therefore, in order to evaluate the effectiveness of the amendments, field trials are required (Ghosh and Maiti 2020; Penido et al. 2019). In this study, we conducted two field experiments in closed mine tailings at Rautuvaara, northern Finland, where we investigated the impacts of BC and CSS application on till soil on the survival and growth of selected plant species, and the potential of BC to reduce the bioaccumulation of metals in plants. We hypothesized that (i) organic amendment (CSS) is needed for vegetation establishment in the tailing cover, (ii) BC application to the cover enhances plant growth further, and (iii) BC retains metals, thereby decreasing their accumulation in plant tissues.

## Materials and methods

### Study site and characteristics of tested materials

The study site is located in the tailings’ repository field at the Rautuvaara enrichment plant in Kolari, Finland (67°29′43.9″N, 23°55′17.3″E) in the northern boreal zone. In addition to tailings (135 ha), there were two water-filled open-pits, an underground mine, a settling pond, an upstream reservoir, and a waste rock facility at the post-mining site (Kivinen et al. 2018).

Mean annual temperature in Kolari (1981–2019) is 0.3°C and precipitation 450 mm, and corresponding values for summertime (June–August) are 12.3°C and 195 mm, respectively. The 2018 summer was warm and dry, and mean summertime temperature and precipitation were 14.3°C and 155 mm in the area. Corresponding values in 2019 were 12.4°C and 151 mm. The native mine soil had not supported plant growth for 30 years since mining ended in 1989. The volume of contaminated sediments at the tailing site was ca. 650,000 m^3^ covering an area of 0.85 km^2^. The acidic water in the pond was reported to contain heavy metals and uranium (Räisänen et al. 2015).

A glacial till (Ti) used in the experiments was dug from the conifer forest soil near the tailings’ repository field. The same till material was also used in actual covering of the entire tailing site in Rautuvaara. The compost material (CSS) that was used consists of sewage sludge composted with peat, wood chips, and sand (Levin Vesihuolto Oy; Sirkka, Finland; Evira acceptance code FIC009-05135/2008NA). A biochar (BC) was made from Norway spruce (*Picea abies*) wood (porosity ca. 1.6 cm^3^ g^−1^) in a batch retort with a holding time of 24 h at 450°C (RKP Ltd., Mikkeli, Finland). Previously, Heiskanen et al. (2020) used the same feedstock materials in their study and published the main physical and chemical characteristics of the tailing soil (Ta0 = non-oxidized and Ta1 = oxidized), Ti, CSS, and BC (Supplementary Material: Tables 1 and 2). Forest mor composed mainly of various mosses, lingonberry (*Vaccinium vitis-idaea*), and bilberry (*Vaccinium myrtillus*) was gently collected as ca. 0.25 m^2^ plates (height ca. 5 cm) also from the nearby coniferous forest.

Two-year-old Scots pine (*Pinus sylvestris*) container seedlings (ca. 150 mm height, collar diameter 4.5 mm) growing in a 0.8-dL peat plug were obtained from Fin Forelia Ltd., Rovaniemi. Willow cuttings (*Salix myrsinifolia*) (200 mm, ⌀ 9–17 mm) from the field of the Natural Resources Institute Finland in Kannus were cut 8 weeks before planting and stored at −3°C. Before planting, cuttings were acclimated to room temperature and kept in water for a week. GREENO PRO road verge seed mixture (S.G.N. Group Ltd.) that is typically used for landscaping purposes in the northern conditions, containing 80% *Festuca rubra*, 6% *Lolium perenne*, 5% *Poa pratensis*, 5% *Festuca brevipila*, 2% *Agrostis capillaris*, and 2% *Trifolium repens*, was used.

### Experimental layouts

**Fig. 1:**
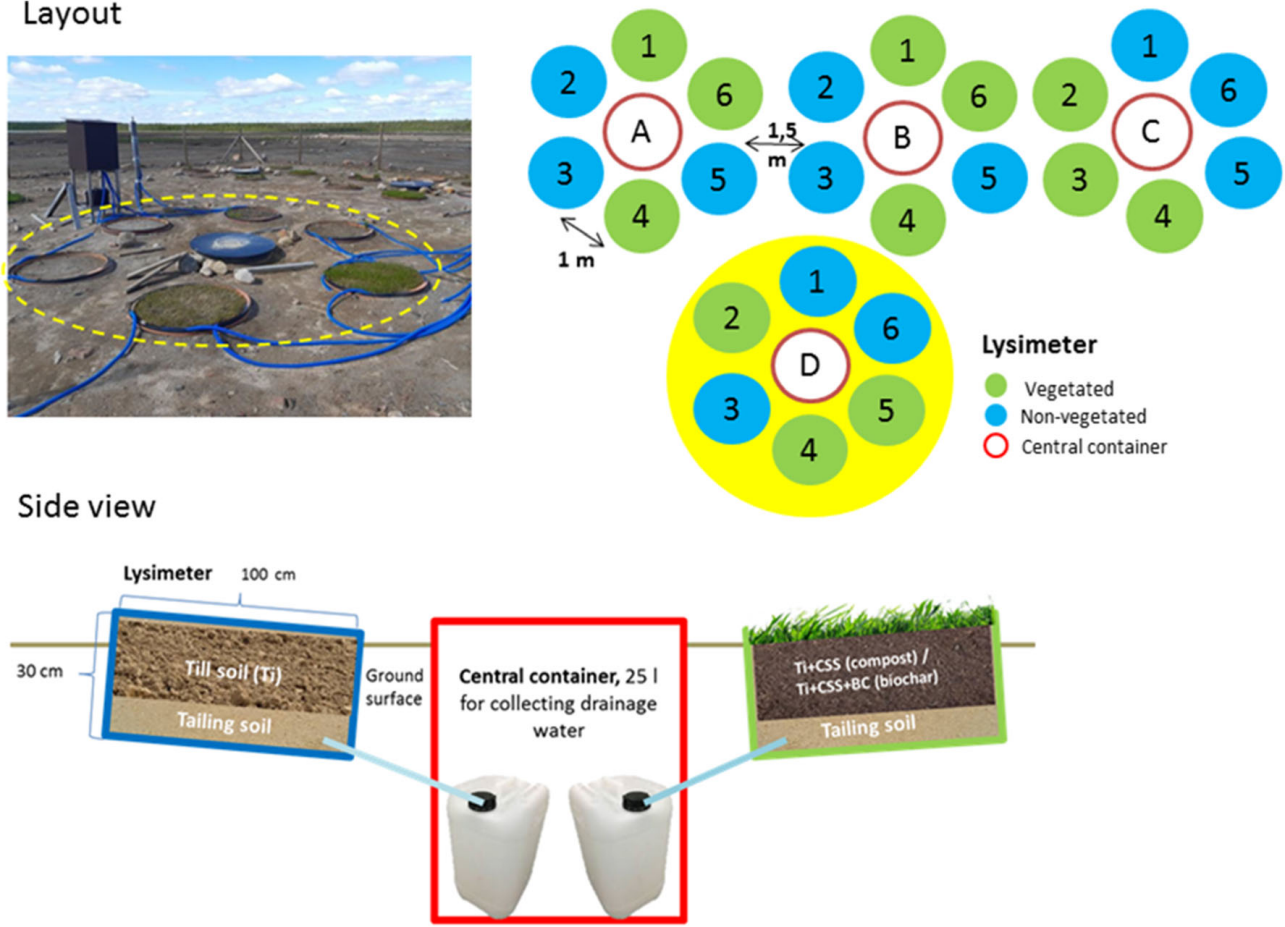
Lysimeter experiment (Exp1) consisted of four units each having six experimental lysimeters and one central lysimeter (in total 24 lysimeters). Each unit contained two lysimeters filled with forest till (Ti), two lysimeters filled with Ti and 10% composted sewage sludge (Ti-CSS), and two lysimeters filled with Ti-CSS applied with 10% biochar (Ti-CSS-BC). Seeds of grass-clover mixture were sown in half of the lysimeters (n=4)

The lysimeter experiment (Exp1) consisted of 24 lysimeters (ø 100 cm, height 30 cm) placed on the Rautuvaara tailing repository area on 13 June 2018. The setup consisted of four lysimeter blocks, each block harboring six lysimeters with a 3-cm rake towards the central container (Fig. 1). Lysimeters were situated at 1.0 m distances from each other at ca. 3 cm depth in tailing soil. The lower 10 cm of each lysimeter was filled with non-oxidized tailing sand shoveled from the experimental site. The top 20 cm of lysimeters was filled with selected test soil types: (1) fresh, transported till (Ti), (2) Ti amended with 10% (v/v) of CSS (Ti-CSS), or (3) Ti amended with CSS (10%, v/v) and BC (10%, v/v) (Ti-CSS-BC) (n=8). Half of the lysimeters were sown with GREENO PRO road verge seed mixture (30 g m^−2^), resulting in four replicates per test soil type treatment both with and without vegetation. Lysimeters were irrigated with 10 L of water after the sowings. Each lysimeter had a 20-mm bottom hole covered with filter cloth (net size 0.2 mm) to prevent the loss of larger particles and blocking of the hose. Drainage water from the lysimeters was directed through plastic hoses (20 mm) to the central container harboring six canisters of 25 L (one for each lysimeter). Central containers were covered with plastic lids. The space between the lysimeters was filled with till so that 2 cm of the lysimeters’ edges remained above the soil surface to prevent rainwater flow from surrounding ground to the lysimeters. Results from leachate waters will be published separately.

In the strip-plot experiment (Exp2), three blocks (each 2 × 3 m) were established at 1.5 m from each other on 14 June 2018 in the Rautuvaara tailing site. Blocks were edged with plastic garden borders (25 cm high) so that the lowest 5 cm of the border was pressed into tailing soil (characteristics of soil: see Supplementary Material Tables 1 and 2) to create 20 cm high edges. Each block was divided in a longitudinal direction with the garden border into two strips (1 × 3 m), which were filled with the test soil types (1) till amended with composted sewage sludge (Ti-CSS, 90/10%, v/v) or (2) till amended with composted sewage sludge and biochar (Ti-CSS-BC, 80/10/10%, v/v/v), resulting in three replicates per test soil type.

Each block was subsequently divided in a latitudinal direction into three strips (1 × 2 m), which were filled (randomly selected) with vegetation types: (A) six pine seedlings + 5 cm layer of forest mor, (B) five willow cuttings + grass seed mixture (30 g m^−2^), and (C) grass seed mixture only (30 g m^−2^), each of three replicates (Supplementary Figure 1). The vegetation of the willow and pine plots mimicked natural vegetation, which has several layers. As a result, each block included one plot (1 × 1 m) from every combination of test soil and vegetation types. Half of the surface of pine plots (0.5 m^2^) was covered with a 5-cm layer of various mosses, dwarf shrubs, and lichens cut into 3-cm pieces. The other side was covered with a transferred forest mor containing a humus layer and vegetation. The forest mor, mosses, etc. were collected from a nearby forest (200 m from the tailings site). These layers, as well as the grasses in the willow plots, represented undergrowth. After establishment and during the initial weeks, each plot was irrigated weekly with 15 L tap water.

### Vegetation success

Vegetation successes of Exp1 and Exp2 were measured two or three times in 2018 (28 June: only pines, 25 July and 29 August: all vegetation types) and twice in 2019 (17 July and 27 August). In both experiments, we measured (1) grass and clover coverage (% based on visual assessment) and height (also willow plots). In Exp2, we additionally measured (2) survival, height, and stem diameter (Mitutoyo CD-15PPX digital caliper) of pine seedlings and (3) germination of new shoots, number of living and dead shoots, and length of living shoots in willow cuttings. In 2019, only two willow cuttings from 30 were alive (Exp2) and thus, no further measurements were done on willows.

In addition, chlorophyll content of plants was measured in both experiments using a portable chlorophyll meter CCM 300 (Opti-Sciences, USA). Chlorophyll measurements were done for ten red fescues, five or ten clovers (depending on the clover percentage on the plot), two needles of each pine, and for each fully grown willow leaf but no more than ten measurements per cutting (for willows only in 2018) per treated plot.

### Sampling and analysis of nutrients and metals

#### Plant sampling and analysis

Plant and soil samples were taken 16 (Exp1) and 14 (Exp2) months after the establishment of the experiments. Grasses and clovers were sampled together as the species were inseparable due to dense plant growth. Furthermore, in Exp1 only aboveground biomass was collected from the whole lysimeter area after the 2nd growing season (October 2019) because lysimeter soil had to remain undisturbed. In Exp2, three 20 cm squares per plot of grasses and clovers were outlined and shoveled (20 cm depth) up (August 2019). In addition, three pine seedlings per plot were carefully removed. Due to the poor survival, willows were not sampled. The roots and aboveground parts of plants were stored separately at +4°C before further analyses.

In the laboratory, fresh and dry mass (60°C, 72 h) of aboveground parts of plants were weighed. Roots of pines and grass-clover mixtures from Exp2 were washed clean under running tap water over a 2-m sieve and the dry mass (60°C, 96 h) of the roots was weighed. Pine roots were pooled to get one sample per plot in order to get enough material for analysis. Plant samples were homogenized with a rotor mill (Retsch ZM 100 Germany, sieve of 0.75 mm trapezoid holes). The aboveground parts of pines contained both needles and stem. Total concentrations of N and C in plant samples were analyzed by Leco TruMac CN-Carbon/Nitrogen Determinator (Leco Corporation, USA). Total concentrations of other nutrients, metalloids, and metals (Al, As, B, Ca, Cr, Cu, Fe, K, Mg, Mn, Na, Ni, P, Pb, S, Zn) were analyzed by the closed wet HNO_3_-H_2_O digestion method in a microwave (CEM MARS 6; CEM Corporation, USA), and iCAP 6500 DUO ICP-emission spectrometer (ICP-AES; Thermo Scientific, UK).

In addition, to get background information about nutrient and metal concentrations of pines, grasses, and clovers in the area, “the control samples” were collected. Pine control samples were collected from nearby conifer forests in Rautuvaara (E67.536980, N23.931308) and Äkäslompolo (E67.578538, N24.073555) and clover samples from a lawn at 20 km distance from the site in Kolari (E67.354623, N23.829231).

#### Soil samples

Soil samples were collected from Exp2 from 0 to 10 and from 10 to 20 cm soil depths by taking three parallel 1 dL samples from each plot and pooling them to get one sample per depth per plot for analysis. Before analysis, the soil was sieved (< 2 mm) to remove larger rock pieces and plant roots. Total concentrations of nutrients and metals were analyzed as previously for plant material except for total nutrients that were digested by the closed wet HNO_3_-HCl digestion method (1:3) in a microwave (CEM MARS 6; CEM Corporation, USA) and iCAP 6500 DUO inductively coupled plasma atomic emission spectrometer (ICP-AES; Thermo Scientific, UK). Concentrations of exchangeable cations and easily soluble phosphorus were analyzed by acid ammonium acetate (pH 4.65) extraction and iCAP 6500 DUO ICP-AES (Thermo Scientific, UK). Concentrations of soluble NH_4_-N, NO_3_-N, and total N were analyzed by 1 M KCl extraction and flow injection analysis (FIA) (Lachat Quickchem 8000, Zellweger Analytics, Milwaukee, MI, USA).

### Statistical analyses

Characteristics of soil and plants (response variables) were modeled with mixed models, mostly with linear mixed models assuming normally distributed observations. Mixed models were used to account for appropriate correlation and variance structure of observations, which leads to more reliable parameter estimates and statistical tests. Depending on the data for the modeled characteristic, one or more random factors (block, plot, plant, and their interaction with fixed factors) with unequal variance of observations (by categories of some fixed factor) were included in the model. A Tukey-Kramer test was used in pairwise comparisons of class means.

In Exp1 with vegetation lysimeters, coverage and height of grasses and clovers were modeled by using the test soil types (Ti, Ti-CSS, or Ti-CSS-BC), time (four dates), and their interaction as fixed factors (n=48). As plant biomass was measured only once, test soil type was the only fixed factor (n=12).

In Exp2 with grass and grass-willow plots, coverage and height of grasses and clovers were modeled by using the test soil types (Ti-CSS or Ti-CSS-BC), time (four dates), and their interaction as fixed factors (n=48). In the height models, also plot vegetation (grass or grass-willow) was included.

In Exp2 with pine plots, we modeled height, proportional growth, and diameter of pine seedling by using the test soil types (Ti-CSS or Ti-CSS-BC), time (five dates), their interaction, and plant height (covariate) as fixed factors (n=174).

In Exp2 with willow plots, we modeled number of leaves, number of living shoots, and length of living shoots in willow cuttings by using the test soil types (Ti-CSS or Ti-CSS-BC), time (2 dates), and their interaction as fixed factors (n=60). Planting diameter of willow cutting was left out from the final model because it was not a significant covariate (P> 0.05). Modelling of number of living shoots (values 0–5) with a negative binomial mixed model or a binary logistic mixed model (after grouping of values) was not successful. Therefore, a linear mixed model was used for an approximate analysis.

In Exp2 with grass and pine plots, we modeled nutrient and metal concentrations and biomass of plants by using the test soil types (Ti-CSS or Ti-CSS-BC), plant type (grass or pine), part of plant (stem or root), and their interactions as fixed factors (n=59, except for biomass n=72).

In Exp2 with all plots, we modeled nutrient and metal concentrations of soil by using the test soil types (Ti-CSS or Ti-CSS-BC), depth of soil (0–5 cm or 5–12 cm), and their interaction as fixed factors (n=12).

The statistical analyses were carried out with the SAS 9.4 software using the FREQ, MIXED, and GLIMMIX procedures.

## Results

Exp1 and Exp2 showed differences based on the test soil type, vegetation success, plant biomass, and accumulation of metals in soil and plants. The results are introduced in this order.

### Vegetation success

In Exp1, the height and coverage of grasses and clovers and the total plant coverage were affected by time and treatment. Also significant interactions between time and treatment were established in both study years (Table 1). Due to these interactions, the data were also tested separately for different sampling times.

**Table 1:** F and P statistics for mixed models effects of time (four sampling events) and test soil types (treatment: Ti-CSS or Ti-CSS-BC) on plant growth in Exp1. Statistically significant (P < 0.05) effects are marked in bold

		DF	F	P
Grass height	Treatment	2	269.04	**<0.0001**
	Time	3	26.27	**<0.0001**
	Time × treatment	6	25.69	**<0.0001**
Grass coverage	Treatment	2	307.76	**<0.0001**
	Time	3	8.05	**0.0007**
	Time × treatment	6	8.21	**0.0003**
Clover height	Treatment	2	353.01	**<0.0001**
	Time	3	31.11	**<0.0001**
	Time × treatment	6	21.66	**<0.0001**
Clover coverage	Treatment	2	13.75	**0.0057**
	Time	3	15.81	**<0.0001**
	Time × treatment	6	7.91	**<0.0001**
Total plant coverage	Treatment	2	1468.44	**<0.0001**
	Time	3	42.82	**<0.0001**
	Time × treatment	6	44.15	**<0.0001**
Plant biomass	Treatment	2	108.81	**0.0009**

**Fig. 2:**
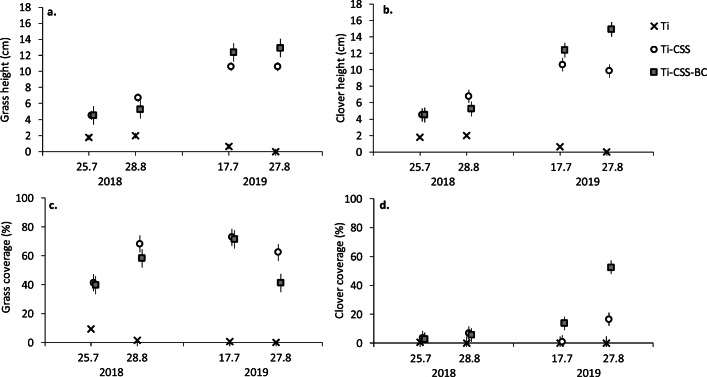
Height and coverage of grasses (**a**, **c**) and clovers (**b**, **d**) sowed on 12.6.2018 and grown in the Rautuvaara experimental lysimeters (Exp1) in fresh till (Ti) or Ti amended with 10% composted sewage sludge (Ti-CSS) or Ti-CSS amended with 10% biochar (Ti-CSS-BC) (model estimated mean ± SE, number of observations n = 48). For statistically significant differences, see Table 2

Four weeks after the establishment of the experiment (July 2018), the lysimeters with Ti as the soil type achieved only 10% total plant coverage that decreased to 0.5–2% over subsequent weeks (August). Total coverage in Ti differed significantly from the corresponding values in Ti-CSS (June and August: 43% and 75%, respectively) and Ti-CSS-BC (42% and 64%, respectively) (Table 2). Also grass and clover height was several times higher in Ti-CSS and Ti-CSS-BC compared to that in Ti only (Fig. 2b, d). Neither the coverage nor height of grasses (Fig. 2a, c) or clovers (Fig. 2b, d) differed statistically significantly between Ti-CSS and Ti-CSS-BC in 2018 (P > 0.05 in each case) (Table 2).

**Table 2:** F and P statistics for mixed models effects of time (four sampling events) and the test soil types (treatments: Ti, Ti-CSS and Ti-CSS-BC) on plant growth in Exp1. Treatment effects shown as pairwise comparisons of the Tukey-Kramer test. Statistically significant (P < 0.05) effects are marked in bold

		Treatment effect			DF	t value	P
Grass height	July 2018	Ti	<	Ti-CSS	19.68	−4.40	**0.0003**
		Ti	<	Ti-CSS-BC	13.37	−2.33	**0.0358**
		Ti-CSS	=	Ti-CSS-BC	15.7	−0.00	1.000
	August 2018	Ti	<	Ti-CSS	19.68	7.60	**<0.0001**
		Ti	<	Ti-CSS-BC	13.37	−2.76	**0.0245**
		Ti-CSS	=	Ti-CSS-BC	15.7	1.20	0.4710
	July 2019	Ti	<	Ti-CSS	19.68	−15.99	**<0.0001**
		Ti	<	Ti-CSS-BC	13.37	−9.97	**<0.0001**
		Ti-CSS	=	Ti-CSS-BC	15.7	−1.40	0.3649
	August 2019	Ti	<	Ti-CSS	19.68	−16.99	**<0.0001**
		Ti	<	Ti-CSS-BC	13.37	−10.98	**<0.0001**
		Ti-CSS	=	Ti-CSS-BC	15.7	−1.85	0.1855
Clover height	July 2018	Ti	<	Ti-CSS	13.34	−5.07	**0.0002**
		Ti	<	Ti-CSS-BC	12.97	−4.52	**0.0007**
		Ti-CSS	=	Ti-CSS-BC	23.56	−0.00	1.000
	August 2018	Ti	<	Ti-CSS	13.34	−8.29	**<0.0001**
		Ti	<	Ti-CSS-BC	12.97	−5.75	**<0.0002**
		Ti-CSS	=	Ti-CSS-BC	23.56	1.26	0.4365
	July 2019	Ti	<	Ti-CSS	13.34	−13.05	**<0.0001**
		Ti	<	Ti-CSS-BC	12.97	−13.56	**<0.0001**
		Ti-CSS	=	Ti-CSS-BC	23.56	−1.47	0.3296
	August 2019	Ti	<	Ti-CSS	13.34	−12.13	**<0.0001**
		Ti	<	Ti-CSS-BC	12.97	−16.37	**<0.0001**
		Ti-CSS	<	Ti-CSS-BC	23.56	−4.24	**0.0013**
Grass coverage	July 2018	Ti	<	Ti-CSS	12.09	−5.49	**0.0001**
		Ti	<	Ti-CSS-BC	12.07	−4.85	**0.0004**
		Ti-CSS	=	Ti-CSS-BC	23.84	0.18	0.9832
	August 2018	Ti	<	Ti-CSS	12.09	−11.43	**<0.0001**
		Ti	<	Ti-CSS-BC	12.07	−9.01	**<0.0001**
		Ti-CSS	=	Ti-CSS-BC	23.84	1.17	0.2540
	July 2019	Ti	<	Ti-CSS	12.09	−12.39	**<0.0001**
		Ti	<	Ti-CSS-BC	12.07	−11.26	**<0.0001**
		Ti-CSS	=	Ti-CSS-BC	23.84	0.18	0.9832
	August 2019	Ti	<	Ti-CSS	12.09	−10.68	**<0.0001**
		Ti	<	Ti-CSS-BC	12.07	−6.56	**<0.0001**
		Ti-CSS	=	Ti-CSS-BC	23.84	2.45	0.0621
Clover coverage	July 2018	Ti	=	Ti-CSS	29.72	−0.47	0.8863
		Ti	=	Ti-CSS-BC	29.72	−0.31	0.9476
		Ti-CSS	=	Ti-CSS-BC	29.72	0.16	0.0866
	August 2018	Ti	=	Ti-CSS	29.72	−1.07	0.5376
		Ti	=	Ti-CSS-BC	29.72	−0.88	0.6577
		Ti-CSS	=	Ti-CSS-BC	29.72	0.20	0.9782
	July 2019	Ti	=	Ti-CSS	29.72	−0.16	0.9866
		Ti	=	Ti-CSS-BC	29.72	−2.15	0.0987
		Ti-CSS	=	Ti-CSS-BC	29.72	−1.99	0.1332
	August 2019	Ti	<	Ti-CSS	29.72	−2.58	**0.0492**
		Ti	<	Ti-CSS-BC	29.72	−8.21	**<0.0001**
		Ti-CSS	<	Ti-CSS-BC	29.72	−5.63	**<0.0001**
Total plant coverage	July 2018	Ti	<	Ti-CSS	12.4	−12.21	**<0.0001**
		Ti	<	Ti-CSS-BC	12.12	−7.89	**<0.0001**
		Ti-CSS	=	Ti-CSS-BC	21.3	0.50	0.8720
	August 2018	Ti	<	Ti-CSS	12.4	−25.63	**<0.0001**
		Ti	<	Ti-CSS-BC	12.12	−15.11	**<0.0001**
		Ti-CSS	=	Ti-CSS-BC	21.3	2.25	0.0905
	July 2019	Ti	<	Ti-CSS	12.4	−25.55	**<0.0001**
		Ti	<	Ti-CSS-BC	12.12	−20.51	**<0.0001**
		Ti-CSS	=	Ti-CSS-BC	21.3	−2.25	0.0905
	August 2019	Ti	<	Ti-CSS	12.4	−27.46	**<0.0001**
		Ti	<	Ti-CSS-BC	12.12	−22.75	**<0.0001**
		Ti-CSS	<	Ti-CSS-BC	21.3	−3.00	**0.0206**
Plant biomass	October 2019	Ti	<	Ti-CSS	3.00	−6.35	**0.0118**
		Ti	<	Ti-CSS-BC	3.00	−15.01	**0.0007**
		Ti-CSS	<	Ti-CSS-BC	5.98	−6.52	**0.0108**

In 2019, total coverage in Ti was below 1% at both measuring times. No statistically significant differences between Ti-CSS and Ti-CSS-BC blocks existed in July sampling for grass and clover height or coverage (Fig. 2a–d). However, in August 2019 the blocks that contained BC had significantly higher total plant coverage, clover height, and clover coverage compared with Ti-CSS (Fig. 2a–d; Table 2; Supplementary Figure 2).

**Fig. 3:**
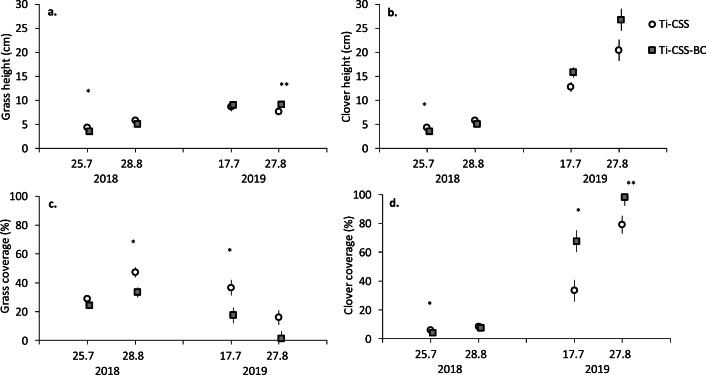
Coverage (**a**, **b**) and height (**c**, **d**) of grasses and clovers (sowed on 12.6.2018) grown at the Rautuvaara experimental site (Exp2) in either fresh till amended with 10% composted sewage sludge (Ti-CSS) or Ti-CSS amended with 10% biochar (Ti-CSS-BC) (model estimated mean ± SE, number of observations n = 48). Statistically significant differences between the treatments among sampling events are marked with asterisks (*P < 0.05, **P < 0.01)

Results from Exp2 were similar to those from Exp1 but the outcomes for the test soils on grass success developed earlier. In Exp2, the effect of soil type on the coverage and height of grasses and clovers varied according to measuring time (Table 3). At the first measuring (July 2018), coverage of clovers and height of grasses and clovers were higher in Ti-CSS than in Ti-CSS-BC (Fig. 3 a–d). In 2018, grass coverage increased between July and August samplings in Ti-CSS from 31 to 47% and in Ti-CSS-BC from 22 to 32%, respectively, differing statistically significantly in August (Fig. 3c). However, in August 2018 neither height of grasses and clovers nor coverage of clovers differed between Ti-CSS and Ti-CSS-BC (Fig. 3a, b, d; Supplementary Table 4).

**Table 3:** F and P statistics from mixed models of the effects of time and the test soil type (treatment) on plant growth in Exp2. Statistically significant (P < 0.05) effects are marked in bold

		DF	F	P
Grass: height	Treatment	1	0.04	0.8372
	Time	3	81.17	**<0.0001**
	Treatment × time	3	4.35	**<0.0393**
Clover: height	Treatment	1	4.82	0.0442
	Time	3	117.83	**<0.0001**
	Treatment × time	3	3.71	**0.0346**
Grass: coverage	Treatment	1	15.50	**0.0011**
	Time	3	20.07	**<0.0001**
	Treatment × time	3	2.82	0.0771
Clover: coverage	Treatment	1	12.98	**0.0017**
	Time	3	138.99	**<0.0001**
	Treatment × time	3	5.43	**0.0092**
Total plant coverage	Treatment	1	0.03	0.8601
	Time	3	842.10	**<0.0001**
	Treatment × time	3	11.39	**0.0004**
Willow: number of leaves	Treatment	1	4.99	**0.0336**
	Time	1	10.56	**0.0030**
	Treatment × time	1	0.14	0.7125
Willow: number of new offsets	Treatment	1	2.00	0.1687
	Time	1	4.50	**0.0429**
	Treatment × time	1	0.24	0.6283
Willow: mean length of offsets	Treatment	1	1.31	0.2618
	Time	1	0.02	0.8950
	Treatment × time	1	2.91	0.0993
Pine: height	Treatment	1	0.00	0.9552
	Time	4	27.40	**<0.0001**
	Treatment × time	4	1.49	0.2253
	Initial length	1	15.43	**0.0005**
Pine: relative growth	Treatment	1	0.58	0.4544
	Time	4	17.22	**<0.0001**
	Treatment × time	4	1.69	0.1792
	Initial length	1	31.32	**<0.0001**
Pine: diameter	Treatment	1	0.07	0.7890
	Time	4	35.65	**<0.0001**
	Treatment × time	4	0.57	0.6894
	Initial length	1	15.06	**0.0005**
Plant biomass	Treatment	1	0.25	0.6213
	Plant species	1	149.5	**<0.0001**
	Plant root vs. shoot	1	0.11	0.7468
	Treatment × species	1	0.44	0.5098
	Treatment × plant structure	1	7.45	**0.0099**
	Species × plant structure	1	17.32	**0.0002**
	Treatment × species × plant structure	1	8.65	**0.0058**

In 2019, the coverage of grasses decreased dramatically in both treatments during summer months (Ti-CSS: from 35 to 17% and Ti-CSS-BC from 18 to 1%) (Fig. 3c). In contrast, the coverage of clovers increased in Ti-CSS from 35 to 78% and in Ti-CSS-BC from 65 to 98% being significantly higher in BC-containing soils in both samplings (Fig. 3d). In 2019, changes in the height of grasses and clovers were similar to those in coverage but the difference was statistically significant only at the last measuring, when BC-containing soils had higher grasses (Fig. 3a; Supplementary Table 4).

**Fig. 4:**
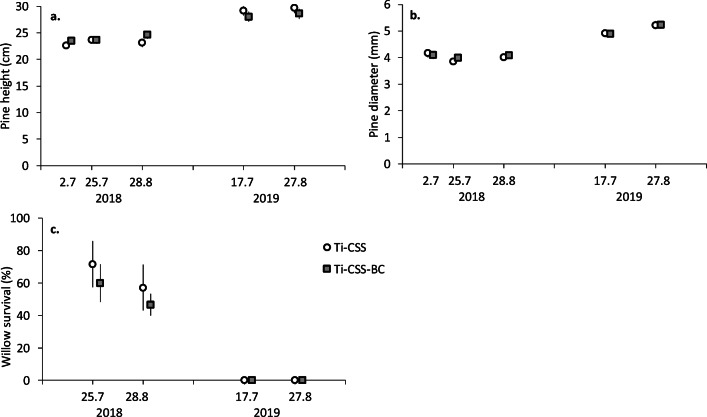
Shoot height and diameter of pine seedlings and survival of willow cuttings during two growing seasons in either forest till amended with 10% composted sewage sludge (Ti-CSS) or Ti-CSS amended with 10% biochar (Ti-CSS-BC) (model estimated mean ± SE, number of observations n = 174 for pine; n = 60 for willow). Each replicate represents a mean for six pine seedlings or five willow cuttings. Statistics in Table 3

Two pine seedlings of 27 died during the 14-month study. Soil amendments had no effect on height (Fig. 4a) or collar diameter (Fig. 4b) of pine seedlings; during two growth periods, their height increased from ca. 200 to 300 mm and diameter from 4 to 5.2 mm.

During the first summer, the survival of willow cuttings declined from 60–75% to 47–58% (Fig. 4c). In the next summer, only two cuttings were alive. Survival was lower in BC-containing soil, but the difference was not statistically significant. Even also the number and mean length of willow cuttings were lower in BC-containing soil, the difference was not statistically significant between the treatments. However, the number of emerged leaves per willow was reduced to 35–45% in the BC-applied soils (Supplementary Table 5). No differences between the treatments in the chlorophyll content of grasses, clovers, pines, and willows were recorded in either Exp1 or Exp2 (Supplementary Table 3).

### Plant biomass

**Fig. 5:**
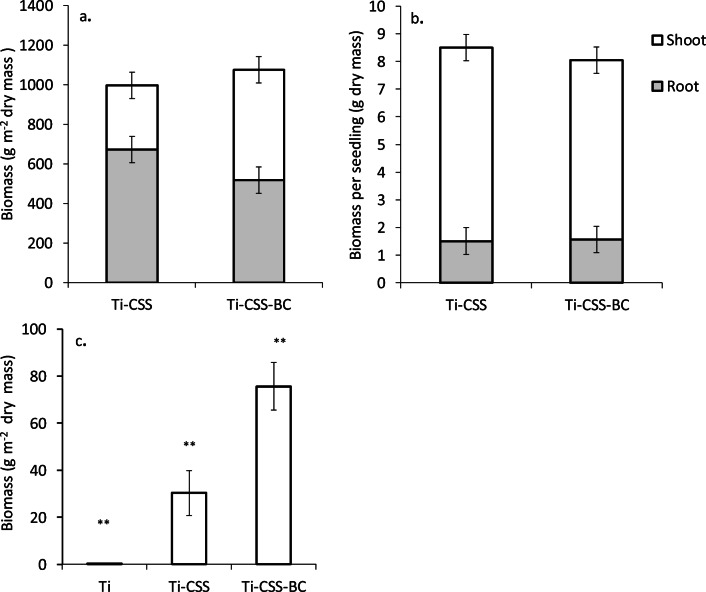
Shoot and/or root biomass of grass-clover mixtures (**a** Exp2, **c** Exp1) and **b** pine seedlings (Exp2) grown for 14 months in either forest till (Ti), Ti amended with 10% composted sewage sludge (Ti-CSS), or Ti soil amended with 10% CSS + 10% biochar (Ti-CSS-BC) (model estimated mean ± SE, number of observations used Exp1: n = 12; Exp2: n = 72). Statistically significant differences between the treatments among sampling events are marked with asterisks (*P < 0.05, **P < 0.01)

Measurements of plant biomass reflected the results of vegetation success. In Exp1, aboveground plant biomass in Ti plots was minimal (Fig. 5c). In Ti-CSS-BC soil, dry weight of plants was 2.5 times higher than for those grown in Ti-CSS (Table 2; Fig. 5c). Also in Exp2, the aboveground grass-clover biomass was on average 71% higher in BC-containing soil (Fig. 5a). In contrast, belowground biomass was 30% lower in BC-containing soils compared with Ti-CSS. However, due to high variation in Exp2, the differences between the treatments were not statistically significant (Table 3; Fig. 5a). In Ti-CSS, on average 69% of grass-clover biomass was allocated belowground in contrast to 49% in Ti-CSS-BC.

After two growth periods, the biomass of pine seedlings was ca. 8–8.5 g (dry mass) with no differences between the treatments (Fig. 5b). Although the depth of the root systems was not measured in this study, according to our visual observations, roots of both grass-clover mixtures and pines had grown through the growing media layer and reached the surface of tailing sands in two growing seasons (Supplementary Figure 3).

### Concentration of metals and nutrients in test soil types

The total concentrations of most elements did not differ between the test soil types (Ti-CSS vs. Ti-CSS-BC) or soil layers (0–5 vs. 5–12 cm) after two growing seasons (Table 4). There were few exceptions: the concentrations of total K, C, Mn, and S were statistically significantly higher in the BC-containing soil. Among the elements in soluble form, the differences in the concentrations of Al (16% reduction in BC-containing test soil), Na (34% increase), and P (24% increase) were statistically significant. Based on the mean value, the concentration of Fe was 1.8–2.9 lower—though not statistically significantly—in the BC-containing test soil. Soil moisture and organic matter content were higher and ash content lower in the BC treatment (Table 4).

**Table 4:** Metal and nutrient concentrations (mg kg^−1^) in the test soil types at the beginning of Exp2 and layers of 0–5 and 5–12 cm after two growing seasons in the mine tailing area. The test soils consist of till and composted sewage sludge without (Ti-CSS) or with biochar (Ti-CSS-BC) (model estimated mean ± standard error). Three columns on the right: P statistics (mixed models) of the effects of test soil (treatment), soil layer (0–5 and 5–12 cm), and their interaction on plant metal and nutrient concentrations. Statistically significant (P < 0.05) effects are marked in bold

	In the beginning			After two growing seasons						
		Ti-CSS^1^	Ti-CSS-	Ti-CSS	Ti-CSS-BC	Ti-CSS	Ti-CSS-BC	Treatment	Soil layer	Treatment × soil layer
			BC^1^	0–5 cm	0–5 cm	5–12 cm	5–12 cm			
Total	Al	10100	9800	8360 ± 551	8990 ± 551	8360 ± 551	9033 ± 551	0.2754	0.9602	0.9649
	As	3.45	3.43	2.93 ± 0.82	2.50 ± 0.57	1.90 ± 0.82	1.47 ± 0.57	0.4947	0.1434	0.9957
	B	1.92	2.16	2.10 ± 0.17	2.71 ± 0.33	2.32 ± 0.17	2.66 ± 0.33	0.1246	0.7529	0.6219
	Ca	5339	5430	6620 ± 438	7010 ± 348	6320 ± 438	6860 ± 348	0.2769	0.5778	0.8578
	Cr	24.6	23.9	27.1 ± 1.56	27.5 ± 1.91	25.7 ± 1.56	26.6 ± 1.91	0.7265	0.5121	0.9117
	Cu	22.7	22.6	15.3 ± 0.43	18.0 ± 1.70	15.5 ± 0.43	17.7 ± 1.70	0.1113	0.9696	0.8888
	Fe	18200	17700	16300 ± 849	18100 ± 849	16500 ± 849	18200 ± 849	0.0755	0.8493	0.9394
	K	1200	1260	**1220 ± 84.3**	**1540 ± 128**	**1280 ± 84.3**	**1490 ± 128**	**0.0426**	0.9614	0.5992
	Mg	2877	2810	3170 ± 332	3250 ± 212	2920 ± 332	3190 ± 212	0.5514	0.5958	0.7582
	Mn	195	196	**208 ± 7.42**	**273 ± 13.9**	**200 ± 7.42**	**242 ± 13.9**	**0.0027**	0.1274	0.3351
	Na	368	361	408 ± 31.1	456 ± 40.7	426 ± 31.1	442 ± 40.7	0.4327	0.9627	0.6903
	Ni	10.1	9.87	12.0 ± 1.80	11.5 ± 0.90	11.2 ± 1.80	10.6 ± 0.90	0.7269	0.6011	0.9614
	P	1450	1440	703 ± 61.3	789 ± 61.3	727 ± 61.3	868 ± 61.3	0.1011	0.4279	0.6580
	Pb	2.47	2.47	1.78 ± 0.09	2.30 ± 0.28	1.70 ± 0.09	2.00 ± 0.29	0.1088	0.3970	0.6186
	S	347	348	**174 ± 50.4**	**238 ± 58.0**	**275 ± 50.4**	**396 ± 58.0**	**0.0236**	**0.0060**	0.3875
	Zn	26.2	27.6	19.5 ± 1.00	31.9 ± 5.15	18.6 ± 1.00	26.0 ± 5.15	0.0518	0.4080	0.5247
	C%	1.16	4.01	**0.44 ± 0.04**	**1.54 ± 0.55**	**0.36 ± 0.04**	**1.93 ± 0.55**	**0.0269**	0.7175	0.5797
	N%	0.17	0.17	<0.08	<0.08	<0.08	<0.08	-	-	-
Soluble	Al	18.1	15.7	**58.1 ± 2.30**	**48.7 ± 2.52**	**60.3 ± 2.30**	**52.2 ± 2.52**	**0.0301**	**0.0388**	0.5054
	Ca	398	440	750 ± 244	639 ± 200	503 ± 244	745 ± 200	0.8403	0.7158	0.3722
	Fe	2.79	2.72	92.0 ± 36.5	32.1 ± 2.97	45.6 ± 36.5	25.3 ± 2.97	0.1968	0.3627	0.4875
	K	35.9	93.4	21.9 ± 1.29	29.3 ± 2.26	28.2 ± 1.29	33.3 ± 2.26	0.3011	**0.0347**	0.4621
	Mg	68.8	69.4	193 ± 75.7	118 ± 52.8	111 ± 75.7	164 ± 52.8	0.8814	0.7732	0.3162
	Mn	4.45	5.07	21.3 ± 5.25	18.7 ± 1.59	12.9 ± 5.25	16.1 ± 1.59	0.9431	0.2218	0.4921
	Na	11.5	11.8	**5.66 ± 1.29**	**8.69 ± 2.52**	**8.93 ± 1.29**	**15.7 ± 2.52**	**0.0370**	**0.0319**	0.3025
	P	0.39	0.46	**6.43 ± 0.87**	**8.50 ± 0.20**	**6.86 ± 0.87**	**10.2 ± 0.20**	**0.0144**	0.1564	0.3543
	S	126	126	83.3 ± 66.5	123 ± 66.5	117 ± 66.5	321 ± 66.5	0.1612	**0.0209**	0.2368
	N	92.8	92.9	9.62 ± 1.58	9.67 ± 1.89	7.48 ± 1.58	6.00 ± 1.89	0.7690	0.0364	0.4479
	NH_4_-N	1.21	1.18	1.09 ± 0.25	1.04 ± 0.13	0.87 ± 0.25	0.92 ± 0.13	0.9905	0.4332	0.8116
	NO_3_-N	90.1	90.1	0.50 ± 0.00	0.50 ± 0.00	0.50 ± 0.00	0.50 ± 0.00	0.3473	0.6999	0.8466
	OM %			**0.97 ± 0.16**	**4.19 ± 0.90**	**1.10 ± 0.16**	**7.45 ± 0.90**	**0.0014**	0.0543	0.0679
	Ash %			**99.0 ± 0.16**	**95.8 ± 0.90**	**98.9 ± 0.16**	**92.6 ± 0.90**	**0.0014**	0.0543	0.0679
	Moisture %			**42.0 ± 2.69**	**62.0 ± 5.72**	**43.7 ± 2.69**	**79.7 ± 5.72**	**0.0009**	0.0763	0.1264

^1^Soluble concentrations of the elements at the beginning of the study are calculated according the values from Supplementary Table 1 by assuming the mixing proportions of 90/10 (Ti-CSS) and (80/10/10) Ti-CSS-BC in volume to produce proportions of 95/5 and 92/5/3 by fresh mass %

Compared to the initial concentrations of the metals and nutrients in different test soil types, the total Cu, N, P, C, and the soluble K and N decreased markedly during two growing seasons (Table 4). In addition, there was a slight decrease in total Al and As concentrations, whereas the total Ca, Cr, and Ni increased slightly and soluble Al, Ca, Fe, Mg, Mn, and P increased significantly during two growing seasons (Table 4).

Some elements accumulated differently according to soil layer. Concentration of total S was 36–39% higher in the 5–12 than the 0–5 cm layer (Table 4). Concentrations of soluble Al, K, and Na were also higher in the 5–12 cm layer and soluble N in the 0–5 cm layer irrespective of the test soil treatment (Table 4).

### Uptake of metals and nutrients in plants (Exp2)

First, we examined whether plants take up nutrients and metals and accumulate carbon (C) differently in Ti-CSS-BC and Ti-CSS soil types. The concentration of total metal (sum of all analyzed metals except Mg) in grasses and clovers (the mixture) in Ti-CSS-BC medium was smaller in shoots by 40% (P = 0.0760) and roots by 29% (P = 0.0494) compared with that in Ti-CSS (Table 5; Supplementary Table 6).

**Table 5:** Metal and nutrient concentrations (mg kg^−1^ dry matter) in plant shoots and roots (pine and grass-clover mixture) after two growing seasons in the mine tailings area covered with 20 cm growing layer containing till and composted sewage sludge without (Ti-CSS) or with biochar (Ti-CSS-BC) (model estimated mean ± SE). Statistically significant (P < 0.05) effects are marked in bold, P < 0.09 italicized. For statistics, see Supplementary Table 6

	Grass				Pine			
	Ti-CSS	Ti-CSS-BC	Ti-CSS	Ti-CSS-BC	Ti-CSS	Ti-CSS-BC	Ti-CSS	Ti-CSS-BC
	Shoot	Shoot	Root	Root	Shoot	Shoot	Root	Root
C%	44.0 ± 1.86	44.5 ± 1.86	26.9 ± 1.91	35.7 ± 1.86	52.4 ± 1.55	52.3 ± 1.55	48.0 ± 1.57	48.5 ± 1.57
N%	1.91 ± 0.12	1.95 ± 0.12	0.59 ± 0.12	0.71 ± 0.12	1.03 ± 0.12	0.98 ± 0.12	0.77 ± 0.13	0.75 ± 0.13
As	3.75 ± 0.66	2.67 ± 0.66	4.54 ± 0.49	3.32 ± 0.46	1.11 ± 0.03	1.07 ± 0.03	3.46 ± 1.01	2.88 ± 1.01
Al	**224 ± 33.4**	**121 ± 33.4**	*6110 ± 646*	*4410 ± 609*	219 ± 20.7	213 ± 20.7	1110 ± 207	905 ± 207
B	15.0 ± 0.88	16.3 ± 0.88	10.7 ± 0.91	11.2 ± 0.88	13.1 ± 0.88	12.6 ± 0.88	6.59 ± 1.25	7.58 ± 1.25
Ca	13200 ± 1630	11500 ± 1630	4750 ± 314	4830 ± 299	2150 ± 246	2400 ± 246	2843 ± 426	3160 ± 426
Cd	0.09 ± 0.03	0.08 ± 0.03	0.22 ± 0.03	0.27 ± 0.03	0.07 ± 0.03	0.07 ± 0.03	0.17 ± 0.03	0.20 ± 0.03
Cr	1.46 ± 0.29	1.01 ± 0.29	42.2 ± 5.05	32.5 ± 4.76	1.59 ± 0.31	1.43 ± 0.31	7.13 ± 1.42	5.59 ± 1.42
Cu	6.11 ± 0.48	5.18 ± 0.48	38.0 ± 2.89	31.2 ± 2.73	4.14 ± 0.40	4.25 ± 0.40	24.2 ± 3.10	21.0 ± 3.10
Fe	*496 ± 79.8*	*296 ± 79.8*	**8020 ± 806**	**5510 ± 760**	243 ± 38.3	273 ± 38.3	1930 ± 305	1600 ± 305
K	18000 ± 902	19000 ± 902	3990 ± 925	5570 ± 902	5430 ± 902	5600 ± 902	5050 ± 1162	4890 ± 1162
Mg	**4780 ± 230**	**5840 ± 230**	1910 ± 245	2190 ± 231	1070 ± 44.9	1050 ± 44.9	1280 ± 77.8	1240 ± 77.8
Mn	421 ± 89.9	267 ± 89.9	341 ± 90.5	386 ± 89.5	**311 ± 28.2**	**227 ± 28.2**	133 ± 46.5	108 ± 46.5
Na	1020 ± 140	1310 ± 140	279 ± 34.8	323 ± 32.8	31.9 ± 2.58	33.0 ± 2.58	98.5 ± 15.5	98.3 ± 15.5
Ni	4.84 ± 0.47	4.45 ± 0.47	29.1 ± 2.96	22.4 ± 2.79	1.45 ± 0.17	1.26 ± 0.17	5.86 ± 1.31	6.95 ± 1.32
P	1580 ± 169	1630 ± 169	2450 ± 175	2170 ± 169	1270 ± 121	1210 ± 121	1460 ± 136	1570 ± 136
Pb	1.68 ± 0.14	1.81 ± 0.32	9.11 ± 0.92	12.3 ± 1.51	1.94 ± 0.56	1.26 ± 0.07	13.2 ± 5.57	9.52 ± 2.74
S	1730 ± 120	1930 ± 120	**1140 ± 127**	**1610 ± 120**	1040 ± 79.6	1050 ± 79.6	1190 ± 120	1290 ± 120
Zn	51.0 ± 7.14	37.4 ± 7.14	57.6 ± 7.30	66.3 ± 7.14	40.7 ± 6.77	41.6 ± 6.77	56.4 ± 7.92	54.9 ± 7.92
Tot metal^1^	*1225*	*753*	**14662**	**10485**	837	777	2289	2722
Tot metal^2^	6005	6593	16572	12675	1907	1827	3569	3962

^1^As + Al + B + Cd + Cr + Cu + Fe + Mn + Ni + Pb + Zn (Mg excluded due to its high level compared with other elements and valid concentration for most plants; Hauer-Jákli and Träkner 2019)

^2^As + Al + B + Cd + Cr + Cu + Fe + Mn + Ni + Pb + Zn + Mg

When the elements were examined separately, only some statistically significant differences were detected between the test soil types. In general, the grass-clover mixture in Ti-CSS-BC had lower concentrations of As, Al, Cr, Cu, Fe, and Ni and in their root tissue in comparison with the Ti-CSS-grown plants. In contrast, concentrations of K, Mg, N, Na, Pb, and S were higher in the grass-clover mixture grown in Ti-CSS-BC media. However, the differences were statistically significant only for Fe and S in roots and for Al and Mg in shoots (Table 5; Supplementary Table 6). Near significant differences were evident for Al in roots and Fe in shoots (Table 5). Compared with Ti-CSS, the roots of grasses and clovers in Ti-CSS-BC had 31% and 28% lower concentrations of Fe and Al, and 40% higher concentration of S. In contrast, in the shoots of grasses and clovers grown in Ti-CSS-BC, the concentration of Mg was 22% higher and Al 46% lower. Also, the concentration of Fe was 40% lower—though not statistically significantly—in the shoots of Ti-CSS-BC-grown grasses. The test soil types showed parallel impacts on metal and nutrient concentration of pines, but only Mn of shoots was significantly lower by 27% in Ti-CSS-BS media (Table 5; Supplementary Table 6).

Secondly, we compared the data within each test soils. Interestingly, the concentrations of the most harmful metals (Al, Cr, Cu, Ni, Pb, As) were 5–40 times higher in the roots compared with the shoots in both plant types irrespective of the test soil type (Table 5). However, many nutrients (N, B, Ca, K, and Mg) were mostly allocated to the shoots. The grasses and clovers accumulated much more Al, Cr, Fe, and Ni in their roots and more N, Ca, Mg, and K in their shoots compared with the pines that grow slower (Table 5; Supplementary Table 6).

Finally, we compared the concentrations in the grasses and clovers grown in our experimental plots with those in the plants that we collected from a nearby area. As, Al, B, Cr, Fe, Mg, Mn, Na, and Ni were higher in the shoots and As, Al, Cd, Cr, Cu, Fe, Mg, Mn, Na, and Ni in the roots of the grass-clover mixture grown at the experimental site in comparison with the background concentrations. The difference was highest for Al (roots: + 10–15 fold), Fe (roots: + 8–11 fold), and Na (shoots: + 56–72 fold). For pine, only slight differences existed between the experimental plants and those collected from nearby forests (Supplementary Table 6).

The total metal concentrations in the composted sewage sludge were far below the threshold values provided by Finland’s environmental authorities (As 25, Cr 300, Cu 600, Hg 1.0, Ni 100, Pb 100, Zn 1500 mg kg (VNa 214/2007)). Similarly, their concentrations in the test soil mixtures in the beginning of the study and after two growing seasons were below the maximum allowed concentrations in soil (As 5, Cr 100, Cu 100, Hg 0.5, Ni 50, Pb 60, Zn 200 mg kg^−1^ (Reinikainen 2007)). Threshold values for Al, Fe, and Mn concentrations for sewage sludge and soil are not published.

## Discussion

Based on earlier findings (e.g., Heiskanen et al. 2020), we expected that organic amendment would be needed for successful vegetation establishment in tailing sites. This assumption was fully confirmed in the field as no regrowth was established in the lysimeters that contained only till (Ti), whereas the addition of composted sewage sludge (CSS) enhanced plant growing significantly. Our second prediction about BC application to test soil for ensuring plant growth in extreme conditions was partly confirmed: the growth of herbaceous plants increased in both experiments. However, BC had no positive effect on survival or growth of pines and willows, which was indicated by the reduced leaf number of willows in BC-containing test soil. In addition, we assumed that BC retains metals, thus decreasing their solubility in the soil, and consequently their concentrations in the plant tissues. This assumption was also partly confirmed as there were clear differences between the test soil types with or without BC. However, the difference was dependent on a metal since the accumulation of Al and Fe in the plant tissues decreased and the concentration of Mg increased significantly when plants were grown in the BC-containing soil. The result also varied based on the plant part and type.

It seems that the response to the application of BC may depend on growth strategies of plants. The results are discussed in more detail below.

### Importance of organic matter

According to visual observations in the Rautuvaara tailing area and our previous greenhouse study (Heiskanen et al. 2020), the early success of vegetation and plant survival are poor in pure mine soil. As we hypothesized, adding a layer of fresh till did not improve the growing conditions—the most used cover material in mine tailings could not maintain the plant cover over the initial years. This result is likely due to low organic matter content, low water holding capacity, and low concentrations of macronutrients in till (Krzaklewski and Pietrzykowski 2002; Tordoff et al. 2000). The growth of herbaceous plants in till can be enhanced however by addition of organic material such as CSS, which increases the amount of nutrients (especially N) and improves soil structure.

The importance of organic material was further stressed by the planted pine seedlings having well-developed root systems. Pine seedlings were potted with peat plugs (organic material) containing some additional nutrients. This supply probably enhanced their growth during the initial growing season in both test soils (with and without biochar). Due to the good start, the roots of grasses, clovers, and pines reached the layer of original mine soil in the second growing period, emphasizing the need for a barrier layer or deeper (>20 cm) soil layer for the tailing cover.

### Improved availability of water and nutrients

The biochar application to the sewage sludge compost and till enhanced the herbaceous plant growth markedly in tailings’ cover. The effect was not, however, seen until the second growing season, most probably due to the warm and dry weather that reduced growth during the year of establishment (i.e., grass coverage and height). Under such conditions, biochar amendment may accelerate soil dryness and render the soil nutrient-poor because biochar can adsorb water molecules and nutrients into its pores and surfaces (Beesley et al. 2011).

On the other hand, we assumed that biochar adsorbed the excess water that arose during the first autumn and following spring, which improved the soil water status in the long term. Thus, the study plants were able to withdraw water in the second growing period (being also dry) after their root systems were better developed, which improved overall plant survival. As neither the total nor the soluble N concentrations differed between the test soil types but the moisture content of the biochar-containing soil type was higher, the result is most probably due to the enhanced moisture conditions in the biochar-containing soil. Referring to Kammann and Graber (2015) and Heiskanen et al. (2020), the pore structure of biochar increased the water holding capacity of till.

Plants often allocate more reserves to their roots when suffering water or nutrient deficiency and assimilates are preferentially exported to the roots. As a result, the root growth is promoted, which helps plants find new water reserves in soil (Chavarria and Pessoa dos Santos 2012). Grasses and clovers growing in the biochar-containing soil seemed to allocate more nutrients to the aboveground biomass. This likely indicates that these fast-growing species were able to allocate the biomass between roots and shoots more evenly in the presence of biochar. Based on Kammann and Graber (2015), they may have achieved this advantage by the development of better root architecture during the drought in 2018.

Interestingly, the N_2_-fixing clovers that obtain nitrogen from the air benefit the biochar application. Mia et al. (2014 and references therein) previously noted that legumes increase aboveground biomass, nodulation, and biological nitrogen fixation when they grow in biochar-supplemented soils (10 t ha^−1^). In our study, clovers obviously benefited more from the enhanced conditions in the soil containing biochar compared with grasses. Our study design was not able to reveal the reason for improved clover growth at the expense of grasses, but it shows that the fast-growing herbaceous species benefit more from the added biochar at least in the short term, since no differences were detected between the biomasses of pines grown on the different test soil types.

Our results are in accordance with earlier studies. In Canada, biochar and sewage sludge in covers enhanced vegetation success on mining waste sites (Dietrich et al. 2017; Drozdowski et al. 2012; Miller and Naeth 2017). In a highly weathered acidic soil, biochar can increase plant growth, efficiency in N and P use, and vegetation cover along with improved seed germination (Anawar et al. 2015; Zhu et al. 2014) because biochar raises soil pH, exchangeable cations, and cation-exchange capacity (Beesley et al. 2011; Park et al. 2011).

The slow-growing pines may gain an advantage from the added biochar in the long run. For example, Pluchon et al. (2014) reported biochar with high P concentration and CEC to increase growth of tree seedlings in northern Sweden. The greater biomass of pine and alder seedlings can partly correspond to an increase in the abundance and diversity of ectomycorrhizal (ECM) morphotypes and more efficient N fixation in root nodules, as Robertson et al. (2012) observed.

Even though biochar increased plant growth in both Exp1 and Exp2, there was a considerable difference in plant biomass between our two studies. This was most probably due to the drier conditions in the insulated lysimeters that received only direct rainwater, in contrast with the experimental blocks in Exp2, which were watered also from the surroundings and by capillary water rising from the deep soil.

Willows showed promising growth in our previous greenhouse trials (Heiskanen et al. 2020) but failed in the field. The reason was most probably linked to the extremely dry summer in 2018. The drought, along with the competition from herbaceous grasses in the field plots, prevented the rooting and growth of the willow cuttings. The root system of the grasses grew fast and higher biomass was yielded. As a result, willows were outcompeted before they were properly rooted.

### Solubility and bioaccumulation of metals

Local residents in northern Finland have expressed their concern about the bioaccumulation that can represent a hazard not only for wild berries and mushrooms (natural products) but also for the fauna, because plants growing on enclosed mine tailings are often food sources for insects, birds, and mammals, especially for reindeer (*Rangifer tarandus domesticus*) (Kivinen et al. 2018). Because metals can bioaccumulate in the food web, selecting the most suitable plant species with low metal accumulation capacity for mine tailings’ restoration is emphasized.

The total concentrations of most of the metals (including some nutrients at low concentrations) in the soils did not differ between the growing media types after two growing periods. However, slightly acidic rainwater and small amounts of CO_2_ and organic acids produced by the roots of the experimental plants presumably increased soil acidity (Adeleke et al. 2017) and further increased the solubility of, e.g., Al and Fe (light metals) from the soil (Sullivan et al. 1988). The primary source of soluble Al, Fe, and Mn was most probably the sewage sludge compost because the concentrations of total Al, Fe, and Mn in the soil did not change, as would occur if there was capillary flow from the mine soil. When we followed the distribution of these metals in the plants, we noted, as was assumed, that the herbaceous plants growing in the biochar-containing soil had lower concentrations of Fe and Al per unit dry weight in their roots and even somewhat lower concentrations in their shoots. However, the total bioaccumulation of metals per hectare in the aboveground biomass was obviously higher in the biochar-containing soils due to the boosted growth.

Masarovičová and Kráľová (2012) argued that uptake and distribution of metals in a plant body depend not only on the concentration of metals but also on their availability. Our study, which shows that using alkaline spruce biochar increases growing media pH and results in smaller increase in solubility (i.e., availability), and as follows the reduced bioaccumulation of metals in plants, agrees with the studies of Fellet et al. (2014), Martins et al. (2018), and Penido et al. (2019). The increased soil pH in biochar-containing soil type is known to reduce the bioavailability of organic and inorganic pollutants (Ali et al. 2017; Fellet et al. 2011, 2014), especially metals such as Pb, Cd, and Zn (Fellet et al. 2014; Martins et al. 2018; Penido et al. 2019). However, increased and neutral effects were reported (Sun et al. 2018 and references therein).

Plant responses to biochar additions to soils may vary based on plant traits and species (Kammann and Graber 2015; Masarovičová and Kráľová 2012). Concentrations of the most harmful metals (Al, Cr, Cu, Ni, and Pb) were significantly higher in the roots of the grasses and clovers (mixture) compared with their shoots, irrespective of the soil treatment. Correspondingly, nutrients (N, B, Ca, K, and Mg) were mostly allocated to the shoots. The herbaceous plants accumulated high concentrations of metals (Al, Cr, Fe, and Ni) in markedly higher amounts in their roots compared with the pine seedlings. However, it has been shown that soil mineral particles tend to attach onto the root epidermis depending on the soil texture and moisture even if roots are washed before drying and weighing (Heiskanen and Rikala 2000). In this light, adsorbed metals on mineral particles of the root surfaces may be interpreted as biochar’s ability to decrease metal bioaccumulation in plant tissues.

Among the observed harmful metals, the acceptable concentration of As in animal feed is 2–10 mg/kg, depending on product type (EC 2002), and the As content in the aboveground parts of the grasses remained at safe levels. There are no similar regulations for Ni, but according to the EFSA risk assessment, the mean concentration of Ni in animal feeds is 9 mg/kg in some products (EFSA 2015). This concentration is two-fold for the Ni concentration of the grass shoots in our research. The results indicate that the experimental plants isolated most of the harmful metals in their roots. Consequently, there is only a slight risk that heavy metals accumulated in the aboveground plant tissues would end up to the feed chain through forage consumption.

We found no differences between treatments in the metal concentrations of shoots and roots of pine, except for Mn. The result can be explained by the slower biomass growth and lower shoot-root ratio of pines compared with grass vegetation. In addition, the peat plug offered nutrients in an easily available form to pine seedlings in both treatments at the beginning of the study. As a result, nutrient uptake was not disturbed by harmful metals that act in a similar manner to nutrients (see Willey et al. 2005). Inevitably, a longer monitoring period is needed to show the long-term effects of the test soil types on the pine seedlings.

## Conclusion

Usability of spruce-derived biochar and composted sewage sludge as a soil type for mine tailings was investigated in two replicated field experiments in the Rautuvaara tailing site in northern Finland. The use of composted sewage sludge proved to be a good landscaping practice in the management of mine tailings. Applying spruce biochar with sewage sludge compost can further increase herbaceous plant growth in harsh mine tailing conditions immediately following tailing cover establishment or in the following season at the latest. Their use can also improve water management, and carbon and nutrient balance of the tailing cover. Adding biochar to transported soil sediment is beneficial because it can decrease bioaccumulation of some harmful metals (Al, Cr, and Fe) that occur in mine soil. Biochar can also help manage metals that sewage sludge may contain.

The use of the recycled products in the covers of mine tailings can also provide environmental solutions for the circular economy by reducing the volume of wastes and giving opportunity to use local forest materials to produce biochar, which have significant impacts on the regional economy and industrial ecosystems. However, because feedstock influences quality of biochar and composted sewage sludge, and quality in turn has a substantial impact on plant success and metal bioaccumulation, feedstock material needs to be taken into account in landscaping, as does the type of mine tailings that affects concentrations of metals in the mine soil.

Furthermore, the growth of roots into mine soil indicates that the depth of the cover material should be greater than the ca. 20 cm layer used in this study, or alternatively an impermeable layer under the soil should be used in order to avoid harmful oxygen transport and diffusion into mine soil. Otherwise, only vegetation of herbaceous species with shallow roots can be established. Using various types of species would improve not only the success of establishment but also the outcome of landscaping through bringing visual and ecological diversity to old mine sites. The issue needs, however, further research. Revegetation of mine tailings can also have other aims, which influence the selection of plant species, such as cleaning-up of contaminated land or producing vegetation for local biochar production.

## Supplementary information

ESM 1 (DOCX 4815 kb)
