# Supplementary material for: Amending mine tailing cover with compost and biochar: effects on vegetation establishment and metal bioaccumulation in the Finnish subarctic
Source: Environ Sci Pollut Res Int. 2021 Jun 20;28(42):59881–98. doi: 10.1007/s11356-021-14865-8 (PMC8542009; doi:10.1007/s11356-021-14865-8)
Supplement: Supplementary file 1 — (DOCX 4815 kb) [file 11356_2021_14865_MOESM1_ESM.docx]

**Amending mine tailings cover with compost and biochar: effects on vegetation establishment and metal bioaccumulation in the Finnish subarctic**

Marleena Hagner^ab*^, Marja Uusitalo^c^, Hanna Ruhanen^d^, Juha Heiskanen^d^, Rainer Peltola^c^, Kari Tiilikkala^ag^, Juha Hyvönen^c^, Pertti Sarala^ef^ and Kari Mäkitalo^c^

*Corresponding author:

email: marleena.hagner@luke.fi

**Supplementary Table 1.** Total nutrients of the growth media components (from combined sample, n=1; extractant aqua regia: HNO3+HCl). Modified from Heiskanen et al. (2020).

|  |  | **Ta0 (non-oxidized)** | **Ta1 (oxidized)** |  |  |  |
| --- | --- | --- | --- | --- | --- | --- |
| **Element** | **Unit** |  |  | **Ti** | **BC** | **CSS** |
| Al | mg/kg | 9760 | 4730 | 8570 | 177 | 38200 |
| As | mg/kg | 101 | 651 | 3.13 | <2.28 | 9.65 |
| B | mg/kg | 5.19 | 11.8 | 1.01 | 8.7 | 19.3 |
| Ca | mg/kg | 50700 | 48100 | 5020 | 7900 | 11400 |
| Cd | mg/kg | 1.29 | 9.59 | <0.07 | <0.2 | 0.178 |
| Cr | mg/kg | 144 | 46.8 | 24.4 | 1.03 | 27.5 |
| Cu | mg/kg | 152 | 199 | 9.46 | 3.94 | 275 |
| Fe | mg/kg | 79300 | 68400 | 16300 | 440 | 53600 |
| K | mg/kg | 4000 | 1010 | 1180 | 2950 | 1620 |
| Mg | mg/kg | 33400 | 19000 | 2820 | 594 | 3960 |
| Mn | mg/kg | 822 | 642 | 187 | 211 | 351 |
| Na | mg/kg | 405 | 280 | 363 | 109 | 483 |
| Ni | mg/kg | 419 | 554 | 9.78 | 1.39 | 16.7 |
| P | mg/kg | 429 | 499 | 482 | 269 | 19800 |
| Pb | mg/kg | 4.33 | 5.43 | 1.92 | <2.28 | 12.8 |
| S | mg/kg | 18800 | 21500 | 46 | 83 | 6070 |
| Zn | mg/kg | 29.9 | 14.4 | 12.9 | 63.1 | 277 |
| C | % DM | 2.73 | 2.26 | 0.161 | 95.1 | 20.2 |
| N | % DM | <0.080 | <0.080 | <0.080 | 0.17 | 1.88 |
| C/N | - | >34.1 | >28.3 | >2.0 | 562.7 | 10.7 |

**Supplementary Table 2**. Extractable nutrients of the growth media components (from combined sample, n=1; extractant BaCl2). ECEC denotes effective cation exchange capacity and BS base saturation. Modified from Heiskanen et al. 2020.

| **Element** | **Unit** | **Ta0** | **Ta1** | **Ti** | **Bc1** | **CSS** |
| --- | --- | --- | --- | --- | --- | --- |
| Al | mg/kg | 1.82 | <0.25 | 4.11 | <1.55 | 238 |
| Ca | mg/kg | 1610 | 2100 | 182 | 1580 | 4500 |
| Fe | mg/kg | 3.27 | 0.396 | 2.65 | <0.309 | 5.36 |
| K | mg/kg | 291 | 95.5 | 13.1 | 1930 | 469 |
| Mg | mg/kg | 1180 | 417 | 41.8 | 59.9 | 582 |
| Mn | mg/kg | 8 | 0.458 | 1.48 | 22.4 | 60.8 |
| Na | mg/kg | 97 | 17.4 | 6.96 | 16.5 | 97.3 |
| P | mg/kg | <0.635 | <0.417 | <0.331 | <2.58 | 1.44 |
| S *) | mg/kg | 3260 | 7360 | 13 | 17.6 | 2270 |
| Ntot **) | mg/kg | 2.59 | 3.65 | 1.91 | 1.35 | 1820 |
| NO_3_ **) | mg/kg | <0.104 | 0.629 | <0.1 | <0.112 | 1800 |
| NH_4_ **) | mg/kg | 1.9 | 1.07 | 1.04 | <1.12 | 5.46 |
| ECEC | cmol/kg | 18.9 | 14.2 | 1.3 | 13.4 | 32.1 |
| BS | % | 100 | 100 | 100 | 100 | 90 |
| pH |  | 7.4 | 7.5 | 5.7 | 9.1 | 6.1 |

**Supplementary Table 3.** Mean chlorophyll concentration (chlr) of plants growing in growth media with and without BC. Pine and willow data are from Exp2. Grass-clover data includes both Exp1 and Exp2.

|  | **Treatment** | **Grass** | **Clover** | **Pine** | **Willow** |
| --- | --- | --- | --- | --- | --- |
| 25.7.18 | Ti-CSS | 1.15 | 0.15 | 0.91 | 1.18 |
|  | Ti-CSS-BC | 1.14 | 1.14 | 0.92 | 1.17 |
| 28.8.18 | Ti-CSS | 1.11 | 1.02 | 1.01 | 1.19 |
|  | Ti-CSS-BC | 1.12 | 1.07 | 0.98 | 1.19 |
| 17.7.19 | Ti-CSS | 0.96 | 1.23 | 0.98 | - |
|  | Ti-CSS-BC | 0.97 | 1.14 | 0.88 | - |
| 27.8.19 | Ti-CSS | 0.95 | 1.20 | 1.02 | - |
|  | Ti-CSS-BC | 0.89 | 1.17 | 1.06 | - |

**Supplementary Table 4.** F and P statistics of mixed models for the effects of time and growth media (treatment) on plant growth in Exp2. Statistically significant (P < 0.05) effects are marked in bold.

|  |  | Treatment effect | | | DF | t Value | P |
| --- | --- | --- | --- | --- | --- | --- | --- |
| Grass height | Time 1 | Ti-CSS | > | Ti-CSS-BC | 9.253 | 5.04 | **0.0008** |
|  | Time 2 | Ti-CSS | = | Ti-CSS-BC | 9.814 | 1.38 | 0.2026 |
|  | Time 3 | Ti-CSS | = | Ti-CSS-BC | 9.941 | -0.32 | 0.7601 |
|  | Time 4 | Ti-CSS | < | Ti-CSS-BC | 2.782 | -2.53 | **0.0336** |
| Clover height | Time 1 | Ti-CSS | > | Ti-CSS-BC | 3.277 | 4.62 | **0.0003** |
|  | Time 2 | Ti-CSS | = | Ti-CSS-BC | 9.999 | 1.40 | 0.1825 |
|  | Time 3 | Ti-CSS | = | Ti-CSS-BC | 10.01 | -2.06 | 0.0563 |
|  | Time 4 | Ti-CSS | = | Ti-CSS-BC | 9.985 | -2.01 | 0.0617 |
| Grass coverage | Time 1 | Ti-CSS | = | Ti-CSS-BC | 9.929 | 2.11 | 0.0530 |
|  | Time 2 | Ti-CSS | > | Ti-CSS-BC | 12.28 | 2.99 | **0.0096** |
|  | Time 3 | Ti-CSS | > | Ti-CSS-BC | 10.16 | 2.52 | **0.0245** |
|  | Time 4 | Ti-CSS | = | Ti-CSS-BC | 9.774 | 2.02 | 0.0632 |
| Clover coverage | Time 1 | Ti-CSS | > | Ti-CSS-BC | 10.00 | 3.16 | **0.0061** |
|  | Time 2 | Ti-CSS | = | Ti-CSS-BC | 10.00 | 0.34 | 0.7391 |
|  | Time 3 | Ti-CSS | < | Ti-CSS-BC | 10.00 | -3.22 | **0.0054** |
|  | Time 4 | Ti-CSS | < | Ti-CSS-BC | 10.00 | -2.23 | **0.0404** |
| Total plant coverage | Time 1 | Ti-CSS | > | Ti-CSS-BC | 4.756 | 2.34 | **0.0337** |
|  | Time 2 | Ti-CSS | = | Ti-CSS-BC | 10.56 | 2.13 | 0.0503 |
|  | Time 3 | Ti-CSS | < | Ti-CSS-BC | 12.17 | -3.49 | **0.0034** |
|  | Time 4 | Ti-CSS | = | Ti-CSS-BC | 4.755 | -2.10 | 0.0540 |

**Supplementary Table 5.** Effect of growth media composed of either forest till soil applied with 1 % composted sewage sludge (Ti-CSS) or Ti-CSS applied with 10% biochar (Ti-CSS-BC) on the number of leaves and number and length of offsets per willow cutting 6 and 10 weeks after planting (mean ± se). Statistical significance (P < 0.05) between treatments among sampling events are bolded (P < 0.05).

| Sampling date | Treatment | Number of leaves | Number of offsets | Mean length of offsets (cm) |
| --- | --- | --- | --- | --- |
|  |  |  |  |  |
| 25.7.2018 | Ti-CSS | **21 ± 3.8** | 2.4 ± 0.4 | 14.6 ± 1.8 |
| 25.7.2018 | Ti-CSS-BC | **14 ± 3.8** | 1.7 ± 0.4 | 11.7 ± 1.7 |
| 28.8.2018 | Ti-CSS | **11 ± 0.4** | 2.1 ± 0.4 | 16.5 ± 2.9 |
| 28.8.2018 | Ti-CSS-BC | **6 ± 0.4** | 1.1 ± 0.4 | 9.4 ± 1.0 |

**Supplementary Table 6.** Left: P statistics (mixed models) of the effects of growth media (treatment), plant species (grass-clover mixture or pine) and plant structure (root or shoot) on plant metal and nutrient concentrations (Ti-CSS compared to Ti-CSS-BB). Right: backround concentrations in the “control plants” collected near tailings site (not included in statistical models).

|  |  |  | Plant structure |  | Treatment x Plant structure | Species x Plant structure | Treatment x Species x Plant structure |  |  |  |  |
| --- | --- | --- | --- | --- | --- | --- | --- | --- | --- | --- | --- |
|  |  | Plant species |  | Treatment x species |  |  |  | Background control | |  |  |
|  |  |  |  |  |  |  |  | Grass |  | Pine |  |
|  | Treatment |  |  |  |  |  |  | shoot | Root | shoot | Root |
| C% | 0.1192 | **0.0001** | **0.0009** | 0.1438 | 0.1128 | 0.0137 | 0.1484 | 46.6 ± 0.06 | 46.6 ± 0.67 | 53.5 ± 0,31 | 50.2 ± 0.60 |
| N% | 0.7563 | **0.0300** | **<0.0001** | 0.4486 | 0.7012 | **<0.0001** | 0.9075 | 3.63 ± 0.05 | 1.75 ± 0.13 | 0.80 ± 0.05 | 0.36 ± 0.00 |
| As | 0.1418 | **0.0102** | **0.0120** | 0.3772 | 0.7183 | 0.1653 | 0.8328 | 1.08 ± 0.00 | 1.08 ± 0.01 | 1.06 ± 0.00 | 1.07 ± 0.00 |
| Al | **0.0373** | **<0.0001** | **<0.0001** | 0.0971 | 0.0622 | **<0.0001** | 0.1436 | 54.6 ± 4.24 | 448 ± 186 | 384 ± 48.4 | 957 ± 153 |
| B | 0.3488 | **0.0257** | **0.0001** | 0.5764 | 0.7365 | 0.3475 | 0.3040 | 8.45 ± 0.41 | 8.26 ± 0.89 | 7.92 ± 2.18 | 3.66 ± 0.11 |
| Ca | 0.6978 | **0.0002** | **0.0025** | 0.4212 | 0.4986 | **0.0010** | 0.5290 | 16900 ± 448 | 5940 ± 78 | 2770 ± 525 | 1340 ± 115 |
| Cd | 0.5014 | 0.1586 | **<0.0001** | 0.8963 | 0.3361 | 0.3326 | 0.6868 | 0.08 ± 0.00 | 0.14 ± 0.03 | 0.15 ± 0.02 | 0.11 ± 0.04 |
| Cr | 0.1165 | **<0.0001** | **<0.0001** | 0.2542 | 0.1562 | **<0.0001** | 0.2862 | 2.94 ± 0.53 | 6.40 ± 0.74 | 2.37 ± 0.75 | 3.75 ± 0.21 |
| Cu | 0.0934 | **0.0008** | **<0.0001** | 0.4491 | 0.1485 | **0.0042** | 0.6723 | 7.84 ± 0.63 | 12.0 ± 0.88 | 13.7 ± 5.30 | 27.5 ± 15.8 |
| Fe | **0.0147** | **<0.0001** | **<0.0001** | **0.0491** | **0.0296** | **<0.0001** | 0.1090 | 176 ± 18.9 | 682 ± 247 | 455 ± 149 | 1929 ± 1100 |
| K | 0.4485 | **<0.0001** | **<0.0001** | 0.4531 | 0.8994 | **<0.0001** | 0.6764 | 22900 ± 375 | 10800 ± 695 | 4160 ± 517 | 2580 ± 460 |
| Mg | **0.0120** | **<0.0001** | **<0.0001** | **0.0070** | 0.1101 | **<0.0001** | 0.1299 | 2896 ± 46.7 | 1433 ± 232 | 696 ± 62.4 | 625 ± 13.0 |
| Mn | 0.2883 | **0.0082** | 0.2132 | 0.9981 | 0.2134 | 0.1118 | 0.4863 | 138 ± 14.2 | 197 ± 47.9 | 383 ± 42.2 | 116 ± 20.5 |
| Na | 0.1132 | **<0.0001** | **<0.0001** | 0.1147 | 0.2332 | **<0.0001** | 0.2378 | 17.9 ± 0.54 | 73.7 ± 14.9 | 35.0 ± 7.17 | 47.6 ± 0.05 |
| Ni | 0.1920 | **<0.0001** | **<0.0001** | 0.0954 | 0.2859 | **<0.0001** | 0.1123 | 2.57 ± 0.21 | 2.41 ± 0.09 | 2.10 ± 0.69 | 2.41 ± 0.38 |
| P | 0.7326 | **<0.0001** | **<0.0001** | 0.5917 | 0.5826 | **0.0059** | 0.0854 | 3220 ± 121 | 2090 ± 116 | 928 ± 56.7 | 644 ± 109 |
| Pb | 0.8715 | 0.8745 | **0.0068** | 0.3111 | 0.9961 | 0.8091 | 0.4111 | 3.93 ± 0.32 | 18.70 ± 2.61 | 1.96 ± 0.43 | 4.59 ± 0.07 |
| S | **0.0387** | **0.0003** | 0.0846 | 0.1107 | 0.2034 | **<0.0001** | 0.5251 | 2030 ± 29.6 | 1340 ± 152 | 617 ± 41.9 | 544 ± 45.0 |
| Zn | 0.7743 | 0.4751 | 0.0058 | 0.8276 | 0.3130 | 0.7333 | 0.2162 | 32.4 ± 2.69 | 100 ± 43.9 | 38.5 ± 4.43 | 17 ± 4.20 |
| Tot Metal^1^ | **0.0297** | **<0.0001** | **<0.0001** | 0.0777 | 0.0517 | **<0.0001** | 0.1377 | 429 | 1478 | 1281 | 3063 |
| Tot Metal^2^ | - | - | - | - | - | - | - | 3326 | 2911 | 1977 | 3688 |

**Supplementary Figure 1.** Experiment (Exp2) established in Rautuvaara tailing site comprised three squares (2 × 3 m) divided into 1 × 1 m plots filled with either Ti-CSS or Ti-CSS-BC and three kind of vegetation (pine seedlings + forest mor, willow cuttings + seeds or seeds only, n=3).

**Supplementary Figure 2. Effect** of 10% biochar amendment on plant growth in lysimeters. Left side: Ti-CSS, right: Ti-CSS-BC.

**Supplementary Figure 3.** Plant roots growing into tailings soil.
